# 3D stimulated Raman spectral imaging of water dynamics associated with pectin-glycocalyceal entanglement

**DOI:** 10.1364/BOE.485314

**Published:** 2023-03-07

**Authors:** Moritz Floess, Tobias Steinle, Florian Werner, Yunshan Wang, Willi L. Wagner, Verena Steinle, Betty S. Liu, Yifan Zheng, Zi Chen, Maximilian Ackermann, Steven J. Mentzer, Harald Giessen

**Affiliations:** 1 4^th^ Physics Institute and Stuttgart Research Center of Photonic Engineering, University of Stuttgart, Pfaffenwaldring 57, 70569 Stuttgart, Germany; 2 Department of Diagnostic and Interventional Radiology, University Hospital of Heidelberg, Im Neuenheimer Feld 420, 69120 Heidelberg, Germany; 3 Translational Lung Research Center Heidelberg (TLRC), German Center for Lung Research (DZL), University of Heidelberg, Im Neuenheimer Feld 156, 69120 Heidelberg, Germany; 4 Laboratory of Adaptive and Regenerative Biology, Brigham & Women’s Hospital, Harvard Medical School, Boston, MA, USA; 5 Institute of Pathology and Department of Molecular Pathology, Helios University Clinic Wuppertal, University of Witten-Herdecke, Wuppertal, Germany; 6 Institute of Functional and Clinical Anatomy, University Medical Center of the Johannes Gutenberg University Mainz, Mainz, Germany

## Abstract

Pectin is a heteropolysaccharide responsible for the structural integrity of the cell walls of terrestrial plants. When applied to the surface of mammalian visceral organs, pectin films form a strong physical bond with the surface glycocalyx. A potential mechanism of pectin adhesion to the glycocalyx is the water-dependent entanglement of pectin polysaccharide chains with the glycocalyx. A better understanding of such fundamental mechanisms regarding the water transport dynamics in pectin hydrogels is of importance for medical applications, e.g., surgical wound sealing. We report on the water transport dynamics in hydrating glass-phase pectin films with particular emphasis on the water content at the pectin-glycocalyceal interface. We used label-free 3D stimulated Raman scattering (SRS) spectral imaging to provide insights into the pectin-tissue adhesive interface without the confounding effects of sample fixation, dehydration, shrinkage, or staining.

## 1. Introduction

Defined as three-dimensional hydrophilic polymer networks, hydrogels provide a promising tool in biomedical applications such as wound healing, drug delivery and tissue repair [1–3]. A particularly intriguing hydrogel is the plant-derived heteropolysaccharide pectin. Structurally, pectin has a high content of partially esterified linear chains of (1,4)-α-D-galacturonic acid residues. Functionally, the pectin chains contribute to the structural integrity of the middle lamella between plant cells by entangling with other pectin chains and cellulose microfibrils [4]. Structurally, pectin is similar to the extracellular surface layer of mammalian cells [5] and pectin chains also entangle with the glycocalyx of visceral organs [6], thereby providing a structural scaffold for wound healing and an opportunity to facilitate tissue repair [7–9].

A distinctive property of polysaccharide polymers, including pectin and the glycocalyx, is the alteration of structural and physical properties with changes in water content [10]. Pectin can demonstrate striking and reversible changes in its physical properties with even small changes in the amount of polymer hydration [11]. The loss of water from a disperse solution of pectin can lead to a change in the physical properties from a viscous liquid to a soft and rubbery gel. With ongoing water loss, the physical properties change from a soft gel to a hard and glassy film. These phase transitions have functional and microstructural implications that remain unclear because of the difficulty in detecting the time-dependent water content in the native pectin polymer.

To identify the water transport dynamics in glass-phase pectin films, we measured water movement in pectin films using 3D stimulated Raman scattering (SRS) microscopy. SRS is an ideal modality as it enables label-free imaging without the need for tissue dehydration, exogenous tracers or chemical fixation [12]. Hence, the addition of chemical fixatives, fluorophores, or tracers is unnecessary. In addition to morphological assessments, quantitative temporal and spectral data evaluation is possible because the SRS signal scales linearly with the concentration of the specimen. Moreover, SRS is free of a non-resonant signal contribution that is present in coherent anti-Stokes Raman scattering (CARS) microscopy [13–17]. In contrast to conventional Raman scattering, SRS exhibits a drastically increased interaction cross-section owing to the simultaneous presence of the pump and Stokes fields. This allows very fast data acquisition speeds up to video rate imaging [18,19], which is ideal to capture the water transport processes.

Using hyperspectral 3D SRS imaging we depict the interface of pectin polymer biomaterial and serosal tissue. This provides, for the first time, quantitative insights into the structural transition zone between pectin and the serosal tissue, as well as the water-dependent entangling phenomenon between a plant-derived polysaccharide network and the mammalian glycocalyx.

## 2. Materials and methods

### 2.1 3D SRS microscope system with active humidity control

**Fig. 1.**
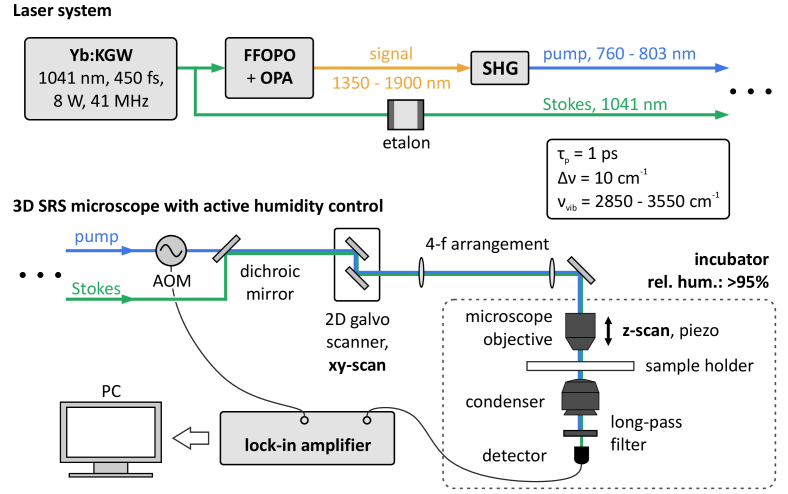
Setup. *Upper panel*: An Yb:KGW oscillator pumps a fiber-feedback optical parametric oscillator (FFOPO). Its signal channel (1350 – 1900 nm) is amplified by an optical parametric amplifier (OPA). Subsequent second-harmonic generation (SHG) provides the tunable Raman pump beam, i.e., 760 – 803 nm. At the same time, the SHG process is used to control the pulse duration via spectral filtering. A part of the oscillator directly serves as the Stokes beam at 1041 nm after spectral filtering using an etalon. Both, pump and Stokes exhibit a pulse duration of ∼1 ps, and thus, 10 cm^-1^ bandwidth. The tuning range allows to access Raman bands between 2850 and 3550 cm^-1^. *Lower panel*: The setup operates in stimulated Raman gain configuration. Therefore, an acousto-optical modulator (AOM) modulates the pump beam at 200 kHz, whereas the Stokes beam is being detected using a single-channel photodiode and a lock-in amplifier. A 2D galvo mirror system allows to scan the sample in lateral direction. Thereby, a 4-f arrangement translates the lateral beam displacement into an angular displacement at the entrance pupil of the microscope objective. Scanning along the beam axis is realized using a closed-loop piezo actuator at the microscope objective mount. The pump beam is spectrally separated from the Stokes beam in front of the detector. The entire microscope is enclosed into an incubator, such that the relative humidity can be kept at high levels in order to prevent sample dehydration, and therefore, shrinkage.

The laser system used for this work is based on a Yb:KGW solid-state oscillator that operates at 41 MHz repetition rate at 8 W average output power und 450 fs pulse duration [20,21], as depicted in Fig. 1. Both, pump and Stokes beams are derived from this oscillator, which intrinsically ensures temporal synchronization.

A part of the oscillator directly serves as the Stokes beam at a fixed wavelength of 1041 nm. Spectral filtering with an etalon tailors its bandwidth to 10 cm^-1^, which results in a pulse duration of ∼1 ps.

In order to obtain a tunable pump beam, and therefore, access to different Raman bands, a fiber-feedback optical parametric oscillator (FFOPO) is employed. Its signal channel (1350–1900nm) is post-amplified by an optical parametric amplifier. Details on this laser system can be found in [22]. Subsequent second-harmonic generation (SHG) employing a 10-mm long periodically-poled lithium niobate (MgO:PPLN) crystal provides the Raman pump beam with a wavelength between 760 and 803 nm. This corresponds to a pump-Stokes detuning range of 2850–3550 cm^-1^.

The SRS interaction manifests as an energy transfer from the pump to the Stokes beam, and thus, as an intensity change thereof. Hence, the SRS signal is intrinsically imprinted on the input wavelength channels, and thus, cannot be retrieved by spectral separation as it is possible with, e.g., coherent anti-Stokes Raman scattering (CARS). Furthermore, the relative intensity change is typically within a range of 10^−7−^10^−5^. In order to measure these small intensity changes, lock-in amplification is employed. This requires intensity modulation of one of the beams, where the respective other beam is detected. In this system the stimulated Raman gain (SRG) of the Stokes beam is detected. Intensity modulation of the pump is realized using an acousto-optical modulator, which operates at 200 kHz modulation frequency. At this frequency the relative intensity noise of the laser system approaches the electronic shot noise limit, and hence, provides the maximum possible signal-to-noise ratio.

Lateral scanning is enabled by a pair of galvanometric mirrors. Hereby, a 4f-arrangement translates the lateral beam displacement into an angular displacement at the entrance pupil of the microscope objective. Axial scanning is realized using a piezoelectric actuator (120 µm travel range), which translates the microscope objective along the beam axis.

The microscope objective (Nikon S Plan Fluor ELWD 60x, NA = 0.7) features a high working distance of 1.8–2.6 mm and is used in air as immersion medium. An oil-immersion condenser with a working distance of 1.6 mm (Nikon C-AA Achromat/Aplanat Condenser, NA = 1.4) is used to collect as much of the transmitted laser light as possible. Since the resulting image quality is solely determined by the microscope objective, the condenser is used in air as immersion medium.

Biological systems typically require specific ambient conditions in order to maintain steady state and prevent the sample from degradation, which especially becomes important during long measurements. In this case, the ambient relative humidity is the critical system parameter, which has to be controlled. Therefore, the entire microscope unit is enclosed in an incubator. A self-designed humidity control system allows to keep the ambient atmosphere at high relative humidity levels close to 100%.

### 2.2 Lateral and axial resolution

Ultimately, the resulting spatial resolution is determined by the volume pixel (voxel) size, where the laser intensities are high enough to drive the SRS interaction. Clearly, this is dictated by the mode matching of pump and Stokes, and the diffraction limit, i.e., the numerical aperture of the microscope objective and laser wavelength. In order to obtain a quantitative figure of merit for the spatial resolution, the nonlinear point spread function (PSF) is measured in lateral as well as in axial direction. Especially, the axial resolution is of interest as the transition zone of pectin placed on top of serosal tissue was analyzed.

**Fig. 2.**
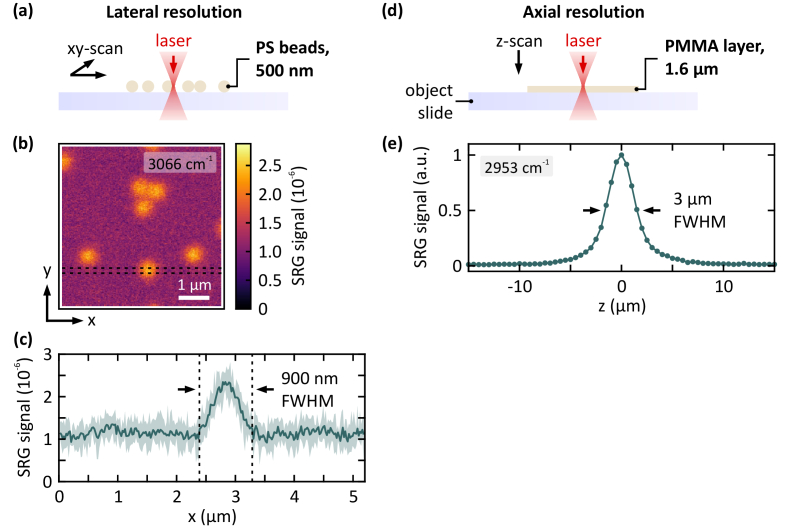
Characterization of the nonlinear point spread function of the SRS microscope in both, lateral and axial, directions. (a) 500-nm polystyrene (PS) beads are imaged in order to determine the lateral resolution. (b) SRS scan at 3066 cm^-1^ with 250 x 250 pixels resolution and 3 ms pixel integration time. (c) Signal cross-sections are extracted from (b) as indicated by the dashed lines. Minimum and maximum values are plotted in light green, the average is given by the solid green line. This yields a full-width at half maximum (FWHM) of 900 nm. (d) A 1.6 µm thick spin-coated PMMA film is scanned along the beam axis. (e) Resulting SRG signal at the PMMA resonance at 2953 cm^-1^, which yields a FWHM of 3 µm in axial direction.

A sample of polystyrene (PS) beads with a known diameter of 500 nm in aqueous solution is used to measure the lateral PSF, see Fig. 2(a). A lateral scan at the Raman resonance of PS (3066 cm^-1^) is depicted in (b). Panel (c) shows SRG signal cross-sections taken along the x-direction, as indicated by the dashed lines. From this, the full width at half maximum (FWHM) is extracted, where the reference signal level is set to zero rather than the background noise level. This results in a conservative value for the width of the lateral PSF of 900 nm and ensures unambiguous separation of spatial features.

The axial resolution is characterized using a spin-coated PMMA film with a thickness of 1.6 µm and by measuring the SRG signal along the beam axis as depicted in Fig. 2(d). The FWHM value amounts to 3 µm. Hence, the axial resolution is sufficient to resolve the interlink range between pectin and tissue, which is expected to be on the order of 10–20 µm.

### 2.3 Sample characterization

**Fig. 3.**
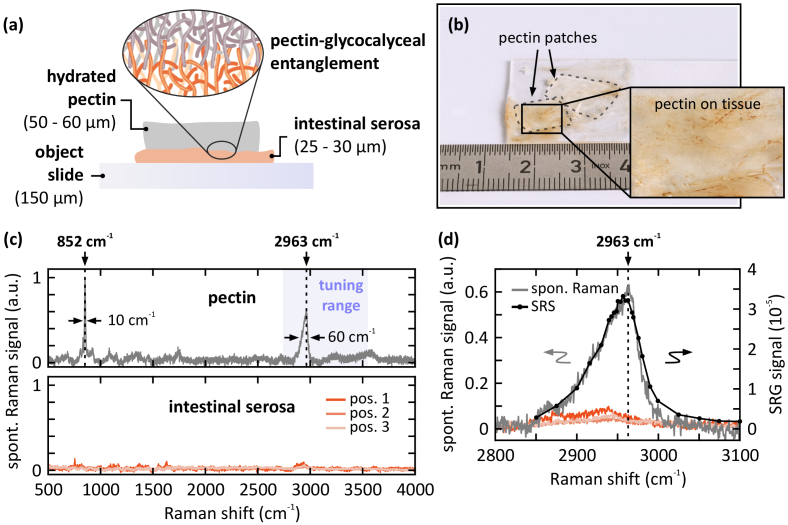
(a) Schematic side view of the sample. Porcine intestinal serosa is covered with a hydrated pectin patch. Inset: schematic of the entanglement between pectin and the glycocalyceal surface of the intestinal serosa. (b) Close-up photograph of the sample, top view. Two pectin patches (dashed lines) are applied to the tissue. (c) Spontaneous Raman spectra of hydrated pectin (upper panel) and intestinal serosa at three different positions (lower panel). Pectin exhibits two prominent peaks at 852 cm^-1^ and 2963 cm^-1^, where the latter is accessible by the laser system (corresponding pump wavelength: 795.6 nm). In contrast, the intestine tissue shows a broad Raman response without any prominent feature. Thus, the peak at 2963 cm^-1^ is ideal to distinguish pectin from the tissue. (d) Zoom-in of the Raman peak of hydrated pectin at 2963 cm^-1^ and the corresponding SRS spectrum.

In order to investigate the interaction of pectin with mammalian serosal tissue, a sample of porcine intestinal serosa is covered with a patch of pre-hydrated pectin, as indicated by the dashed lines in Fig. 3(b). Hydrated pectin is much more transparent for pump and Stokes wavelengths than the intestinal tissue. This, in combination with its structural homogeneity, minimizes laser wavefront distortions during propagation. Since SRS is a phase-sensitive process, these wavefront distortions deteriorate the signal level. Therefore, the pectin polymer was placed on top of the tissue, as depicted in Fig. 3(a), in order to maintain high imaging quality in deeper sections of the sample.

First, the Raman responses of pectin as well as of the intestinal tissue were characterized. Therefore, broadband *spontaneous* Raman spectra are acquired, as depicted in Fig. 3(c). Pectin exhibits two prominent Raman resonances, located at 852 cm^-1^ in the fingerprint spectral region, and at 2963 cm^-1^ in the C-H stretch region (upper panel). The shaded area indicates the accessible spectral range of the laser system. The intestinal serosa, on the other hand, does not exhibit any prominent specific spectral features (lower panel). Thus, the Raman peak at 2963 cm^-1^ is being used to track the pectin concentration. As a cross-check, a *stimulated* Raman spectrum of the pectin resonance is additionally acquired, as depicted in Fig. 3(d). Both, the resonance position as well as the spectral shape agree well. Note, that the pectin resonance partially overlaps with the protein signal, which is located around 2950 cm^-1^.

## 3. Results and discussion

### 3.1 Hydrophilicity of the hydrogel network

Hydrating the dry pectin prior to applying it onto pleural tissue is required to initiate pectin-glycocalyceal entanglement *in vitro*. *In situ*, the secretory lubricating properties of mesothelial cells facilitate the initial hydration process and microstructural entanglement. This hydration process and interrelated water transport dynamics within the pectin polymer are not yet well understood. Here, we use SRS to determine the hydrophilicity by quantitatively monitoring the spatial water content over time within the pectin hydrogel network.

Hereby, two mechanisms are investigated, namely, the water absorption from a humid ambient atmosphere, and the water transport within the pectin polymer by direct contact with the liquid water. Both mechanisms are involved in the hydration process of the pectin polymer in biomedical use, and hence, it is important to determine their respective time scales.

**Fig. 4.**
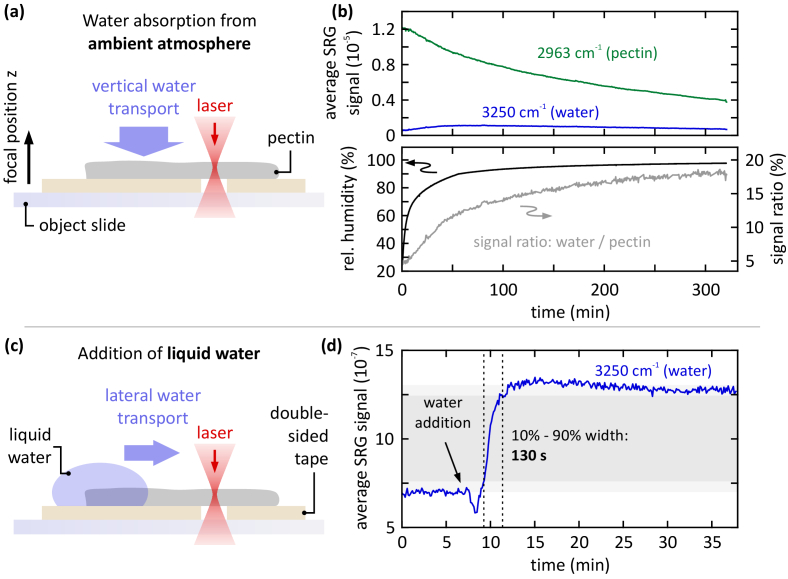
Water absorption and transport dynamics in pectin are measured by tracking the SRS signal levels at the water and pectin resonance. Hereby, the pump-Stokes detuning is automatically switched between 2963 cm^-1^ and 3250 cm^-1^. Pump power: 5 mW, Stokes power: 4.5 mW. The temperature is constant at 24 °C. (a) Water absorption from the ambient atmosphere at high relative humidity. (b) Upper panel: individual signal traces for pectin and water. Lower panel: signal ratio between water and pectin, as well as the ambient relative humidity in the microscope incubator. In this case, hydration takes place on the time scale of ∼5 h. Each data point corresponds to the average signal obtained from a 50-by-50 µm^2^ FOV with 20-by-20 px^2^. 8 s acquisition time per data point, 4 s effective integration time. (c) Liquid water is applied to the pectin patch and the water transport is tracked in lateral geometry. (d) For lateral water transport, the hydration process takes place on the time scale of ∼2 min (10% - 90% width, indicated by dashed lines and grey shaded area). Each data point corresponds to the average signal obtained from a 50 x 5 µm^2^ FOV with 50 x 5 px^2^, where the water transport is directed along the short edge of the FOV. Hence, the corresponding diffusion coefficient amounts to $D=\langle x^{2}\rangle /2t\;∼\;10^{-13}$ m^2^/s. 5 s acquisition time per data point, 2.5 s effective integration time.

For practical reasons the water absorption from ambient atmosphere is measured in vertical geometry, whereas the water transport from direct contact is measured in horizontal geometry, as depicted in Fig. 4(a) and (c). Since the hydration takes place directionally, e.g., from the top as shown in Fig. 4(a), the pectin polymer bends due to the expanding hydrogel network. In order to avoid motion artifacts by pectin bending during the measurement, the pectin patch was fixed to the object slide by double-sided tape with an aperture for the laser to be transmitted, see the schematics in Fig. 4.

The SRS signal scales linearly with the oscillator concentration, i.e., the water concentration in this case. However, it is necessary to also track the pectin signal channel simultaneously as the pectin polymer expands during hydration, and therefore, the overall oscillator concentration changes. Therefore, the relative signal level between water and pectin is the significant measure, as shown in the lower panel of Fig. 4(b). The laser focus is positioned at a constant z-position beneath the interface between pectin and the air. Notably, the focal position relative to the interface changes over time due to the volume expansion of the pectin polymer.

The relative SRS signal follows the ambient relative humidity (black line) with a delay, which is caused by the slow water absorption. The overall absorption dynamics spans across ∼5 h. Hereby, a relative mass change of 24% is measured between t = 0 and the steady state at t = 335 min.

The water transport dynamics is measured by applying a liquid drop of water to a dry patch of pectin polymer, which immediately starts to absorb the liquid water (real-time video footage of this process can be found in the supplementary material). A well-defined waterfront passes through the field of view (50 × 5 µm, details can be found in Fig. S2(b) in the Supporting Information) along its short edge, i.e., across a distance of 5 µm. Hereby, the SRS signal is recorded at the water resonance at a constant z-position within the pectin. In this measurement, only the water signal is monitored, since only the relative change of the local water content is of interest. As determined from the 10% – 90% width, the waterfront traverses the field of view within 130 s. Assuming simple diffusion, the corresponding diffusion coefficient amounts to $D=\langle x^{2}\rangle /2t\;∼\;10^{-13}$ m^2^/s at 24 °C, where $\left\langle x^{2}\right\rangle$ denotes the average square of the traversed distance within the time *t* [23]. Thus, the water transport dynamics within the pectin is ∼2 orders of magnitude faster than the water absorption from the ambient atmosphere. This large difference between these two time scales also manifests in the signal ratio being independent of the z-position within the pectin, see Fig. S1 in the Supporting Information. Hereby, no temporal delay between the signal ratio curves at different z-positions is observed. Furthermore, no structured water channels in the pectin hydrogel are visible, as depicted in Fig. S2 in the Supporting Information. Hence, the water transport takes place homogeneously on a molecular scale or, on the other hand, potential water channels are well below the diffraction limit.

### 3.2 Pectin-glycocalyceal transition zone

**Fig. 5.**
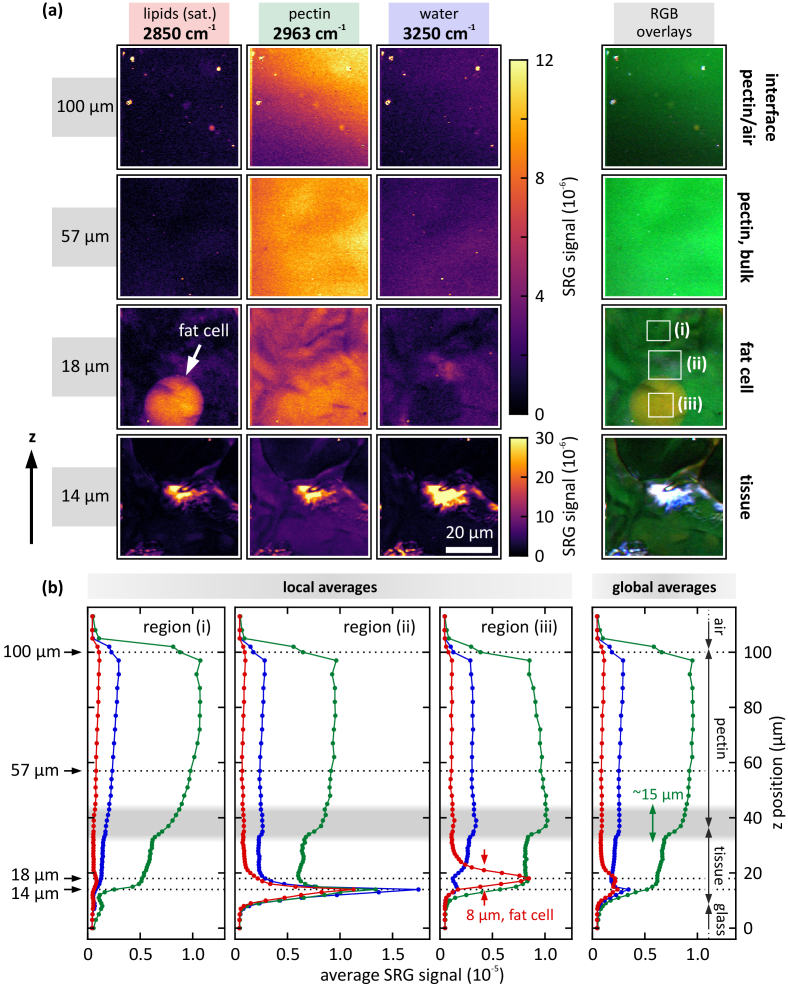
*Composition of the pectin mesothelial interface*. (a) Hyperspectral 3D SRS scan through the sample, measured at three Raman bands: 2850 cm^-1^ (saturated lipids), 2963 cm^-1^ (pectin), and 3250 cm^-1^ (water). 2D scans at z = 14, 18, 57, and 100 µm are depicted as well as RGB overlays thereof, where each of the three resonance positions is encoded as one color channel. The pleural tissue (z = 14 µm) exhibits structures in the pectin channel, which mainly originates from the close-by protein band at 2950 cm^-1^. At z = 18 µm a mesenteric fat cell with a diameter of 25 µm is revealed at the resonance of saturated lipids. The pectin bulk material at z = 57 µm causes a homogeneous signal distribution, which extends up to the pectin-to-air interface at z = 100 µm. (b) Signal cross-sections along the z-axis for each of the three Raman bands. Local averages are calculated for the rectangular regions (i) – (iii) as indicated in (a), global averages take into account one entire image for each data point. As shown by the green curves the pectin layer exhibits a thickness of ∼65 µm, which extends from z = 35 – 100 µm. Region (iii) shows the axial dimension of the mesenteric fat cell, i.e. 8 µm. On average, the interlink length (gray shaded area) between tissue and pectin amounts to ∼15 µm. Imaging parameters: 12 mW pump power, 4.5 mW Stokes power, 50 x 50 µm^2^, 150 x 150 px^2^, 2.5 ms pixel dwell time, 1 ms integration time per pixel, ∼1 min acquisition time per image. Ambient conditions: 25 °C, 96% relative humidity.

In the following, the structural transition and the interlink length between the pectin hydrogel and intestinal serosa tissue is measured using 3D SRS imaging. Therefore, the sample is prepared as shown in Fig. 3 and placed in the incubator ∼1.5 h before the SRS scan is started. This ensures that the water uptake from the ambient atmosphere is equilibrated, and thus, the sample is in a steady state. A range of ∼100 µm in z-direction is scanned, where the zero position is set to be within the object slide directly beneath its interface to the intestinal tissue. Since hydrated pectin is highly transparent for the employed wavelengths, and the intestinal serosa tissue is only ∼25 µm in thickness, no corrections for depth-dependent signal attenuation are necessary. Figure 5(a) depicts a part of the hyperspectral data set. In particular, 2D cross-sections at z = 14, 18, 57, and 100 µm for three Raman resonances are shown, each image covers a field of view of 50 × 50 µm^2^. For each z-position, the three spectral channels (unsaturated lipids at 2850 cm^-1^, pectin at 2963 cm^-1^, and water at 3250 cm^-1^) are combined into an RGB overlay, where each spectral position corresponds to one color channel. A colorblind-friendly version of the overlay images can be found in Figure S3 in the Supporting Information.

With the pectin placed on top of the tissue, in order to evaluate the signal transitions and the interface, the average SRS signals are plotted against their respective z-position, see Fig. 5(b). In particular, each 2D scan is averaged across the entire field of view (right panel). Locally resolved averaging is done according to regions (i) – (iii) as indicated in the RGB overlay at z = 18 µm in panel (a). The center area of the field of view at z = 14 µm exhibits a strong signal, which is independent of the spectral position. This spectrally independent signal is usually caused by parasitic cross-phase modulation (XPM) between pump and Stokes, which enters the same detection channel as signals, that arise from SRS interaction. Therefore, XPM intrinsically cannot be rejected by the detection unit. At z = 18 µm a fat cell with a diameter of ∼25 µm is revealed at the resonance of saturated lipids (2850 cm^-1^). Its thickness amounts to 8 µm as extracted from the axial signal trace for region (iii), see the red curve in Fig. 5(b). These dimensions match the typical size of fat cells. In contrast, the fat cell does not exhibit any substantial signal at the water resonance (3250 cm^-1^). Hence, this demonstrates an off-resonant case for the Raman transitions in saturated lipids. Furthermore, the pectin channel at z = 18 µm exhibits a strong signal, which can be traced back to the overlapping protein resonance at 2950 cm^-1^, see supplementary Fig. S4. In the pectin bulk material, the SRS signal level is clearly dominated by the resonance at 2963 cm^-1^. Here, the signal is homogeneously distributed across the field of view, as exemplarily shown at z = 57 µm. As expected, the water channel also exhibits a homogeneous signal distribution, caused by water molecules being incorporated within the pectin polymer. At z = 100 µm the pectin polymer interfaces the air. Apparently, the pectin surface is slightly tilted with respect to the object slide, which causes the signal gradient in the image.

From the axial signal traces in Fig. 5(b) the interface between pectin and the glycocalyx as well as the pectin-glycocalyceal entanglement length can be extracted. Between z = 35 and 97 µm all signal channels, including protein and unsaturated lipids (see supplementary Fig. S4), exhibit constant signal levels. Hence, this range contains the structurally homogeneous pectin layer. In contrast, within the intestinal tissue the signal levels vary more strongly due the more inhomogeneous distribution of the chemical constituents. The zone of pectin-glycocalyceal entanglement can be extracted from the width of the signal transition from the tissue to the pectin hydrogel, as indicated by the gray shaded area. In particular, the interlink length amounts to ∼15 µm on average, which is well in line with recent data we derived from high pressure freezing transmission electron microscopy, which estimates the thickness of the mammalian visceral glycocalyx to a thickness of ∼13 µm [5]. Clearly, this value varies locally as evident from the first three plots in panel (b).

Additional to unsaturated lipids, pectin, and water, the signal is also acquired for proteins at 2950 cm^-1^ as well as for unsaturated lipids at 3015 cm^-1^. Both resonances are included in Fig. S4 in the Supporting Information. In particular, the protein resonance is located close the pectin resonance.

The total acquisition time for the entire data set amounts to 5 h 45 min. Hence, the incubation unit of the SRS system is crucial to ensure sample stability over this period of time. The absolute positioning drift of the sample due to dehydration across the measurement time is determined to a very low value of 1.5 µm/h on average, details can be found in the Supporting Information in Fig. S5. This low drift also suggests that the incident laser does not have any substantial impact on the water content.

### 3.3 Limitations of this study

Technical restrictions impose limitations on this study, which is discussed here. For instance, the signal-to-noise ratio (SNR) ultimately determines the acquisition time. The SNR scales linearly with the laser power in each of the two wavelength channels. However, the laser power is limited to several milliwatts, otherwise photo damage as well as dehydration due to local heating occur in the sample. The impact of the incident laser on the water content could be quantified using hydrated pectin in a steady state, i.e., all processes associated with material-specific water transport dynamics are equilibrated. A power-dependent measurement of the signal ratio between water and pectin would provide a figure of merit for laser power-dependent effects on the water content. Regions closer to the surface are expected to be more susceptible to dehydration than the bulk material. Due to the restricted laser power level, very fast sample dynamics, such as early aspects of the pectin-glycocalyceal entanglement process itself may not be resolved temporally, or only with limited spatial resolution and/or with a limited number of spectral positions. Resolving the entanglement process is particularly challenging, as it additionally involves a volume change, and therefore, a non-steady z-position. For a further in-depth understanding of these specific aspects of the process, we plan to correlate the SRS-derived data to non-time resolved, but very spatially resolved electron microscopy in the future.

As SRS is phase-sensitive, the sample thickness is limited by its scattering properties. Especially inhomogeneous biological samples tend to deteriorate the wavefronts, such that the sample thickness is limited to a few tens of microns. This was sufficient for our study, because our focus was on the superficial surface layer of the organ, but may limit wider applications in other studies that involve thick tissues.

## 4. Conclusion

In this work we investigated the hydrophilicity of a plant-derived pectin hydrogel using 3D stimulated Raman scattering microscopy. The two hydration mechanisms, water absorption from ambient atmosphere and hydration by direct contact with the liquid water, exhibit largely different time scales, separated by nearly two orders of magnitude. These findings are of importance especially for wound healing, since the structural and physical properties of this hydrogel network are strongly dependent on its water content. In particular, optimal adhesive properties of pectin on organ surfaces requires a correct hydration level. Furthermore, we used 3D stimulated Raman scattering imaging in order to quantify the interlink length of hydrated pectin and mammalian glycocalyx.

In the future we plan to measure the volume expansion of the pectin hydrogel associated with hydration. Thereby, the position of the interface between pectin and the air can be tracked with high precision using the z-scan capability of our SRS microscope. Our study will provide new quantitative insights into the structural transition zone between pectin and the serosal tissue, as well as the water-dependent entangling phenomenon between pectin and the mammalian glycocalyx, which can further enable medical applications such as serosal wound healing and visceral tissue repair.

## Data Availability

Data underlying the results presented in this paper are not publicly available at this time but may be obtained from the authors upon reasonable request.

## Supplemental document

See Supplement 1 for supporting content.
