# Supplementary material for: 3D stimulated Raman spectral imaging of water dynamics associated with pectin-glycocalyceal entanglement
Source: Biomed Opt Express. 2023 Mar 7;14(4):1460–71. doi: 10.1364/BOE.485314 (PMC10110326; doi:10.1364/BOE.485314)
Supplement: Supplementary file 1 [file boe-14-4-1460-s001.pdf]

Supplemental Document

Biomedical Optics EXPRESS

3D stimulated Raman spectral imaging of water dynamics associated with pectin-glycocalyceal entanglement: supplement

MORITZ FLOESS,[1,*] TOBIAS STEINLE,[1] FLORIAN WERNER,[1] YUNSHAN WANG,[1] WILLI L. WAGNER,[2,3] VERENA STEINLE,[2] BETTY S. LIU,[4] YIFAN ZHENG,[4] ZI CHEN,[4] MAXIMILIAN ACKERMANN,[5,6] STEVEN J. MENTZER,[4] AND HARALD GIESSEN[1]

[1] *4th Physics Institute and Stuttgart Research Center of Photonic Engineering, University of Stuttgart, Pfaffenwaldring 57, 70569 Stuttgart, Germany*
[2] *Department of Diagnostic and Interventional Radiology, University Hospital of Heidelberg, Im Neuenheimer Feld 420, 69120 Heidelberg, Germany*
[3] *Translational Lung Research Center Heidelberg (TLRC), German Center for Lung Research (DZL), University of Heidelberg, Im Neuenheimer Feld 156, 69120 Heidelberg, Germany*
[4] *Laboratory of Adaptive and Regenerative Biology, Brigham & Women's Hospital, Harvard Medical School, Boston, MA, USA*
[5] *Institute of Pathology and Department of Molecular Pathology, Helios University Clinic Wuppertal, University of Witten-Herdecke, Wuppertal, Germany*
[6] *Institute of Functional and Clinical Anatomy, University Medical Center of the Johannes Gutenberg University Mainz, Mainz, Germany*
[*] *moritz.floess@pi4.uni-stuttgart.de*

This supplement published with Optica Publishing Group on 7 March 2023 by The Authors under the terms of the Creative Commons Attribution 4.0 License in the format provided by the authors and unedited. Further distribution of this work must maintain attribution to the author(s) and the published article's title, journal citation, and DOI.

Supplement DOI: https://doi.org/10.6084/m9.figshare.22181725

Parent Article DOI: https://doi.org/10.1364/BOE.485314

3D stimulated Raman spectral imaging of water dynamics associated with pectin-glycocalyceal entanglement: supplemental document

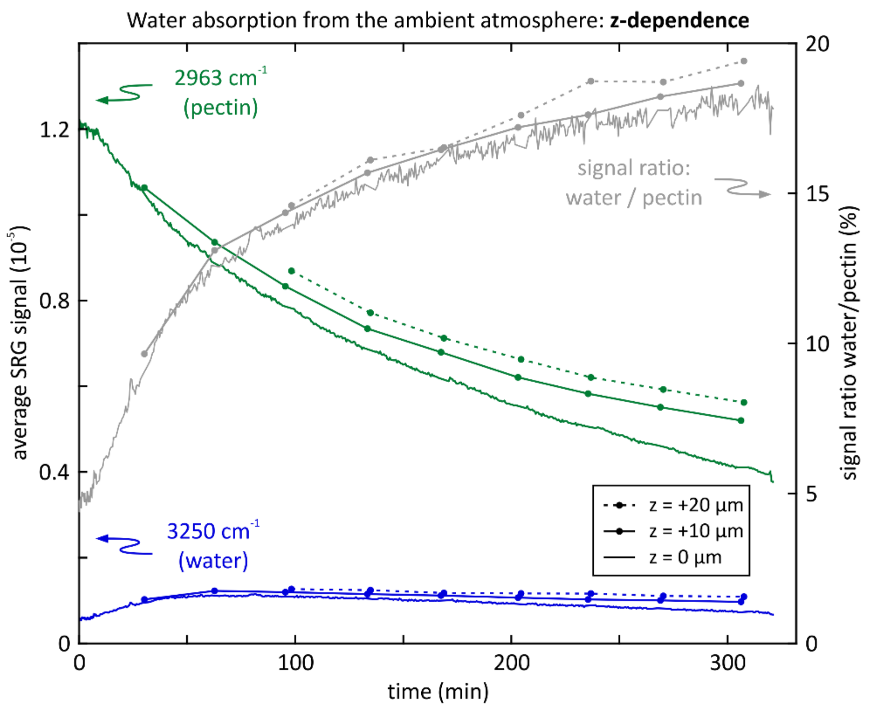

Figure S1. Additional z-dependence of the water content within the pectin polymer. Solid line: data shown Fig. 4 of the main manuscript, measured at a constant z-position, which is used as the reference position. Additionally, the signal levels are measured 10 µm and 20 µm above the reference level.

In Fig. S1, both individual signal levels, water and pectin, decrease for lower z-positions, whereas their signal ratio does not change. Hence, this signal decrease does not originate from a z-dependent water content within the pectin polymer. Rather, it is caused by a laser power drop due to the absorption and/or scattering and by wavefront distortions arising from inhomogeneities in the pectin bulk material. The latter deteriorates the phase-sensitive SRS signal level.

Since the water transport dynamics within the pectin are much faster than the absorption from the ambient atmosphere (Fig. 4 in the main manuscript), no temporal shift between the signal ratio curves at different z-positions is observed.

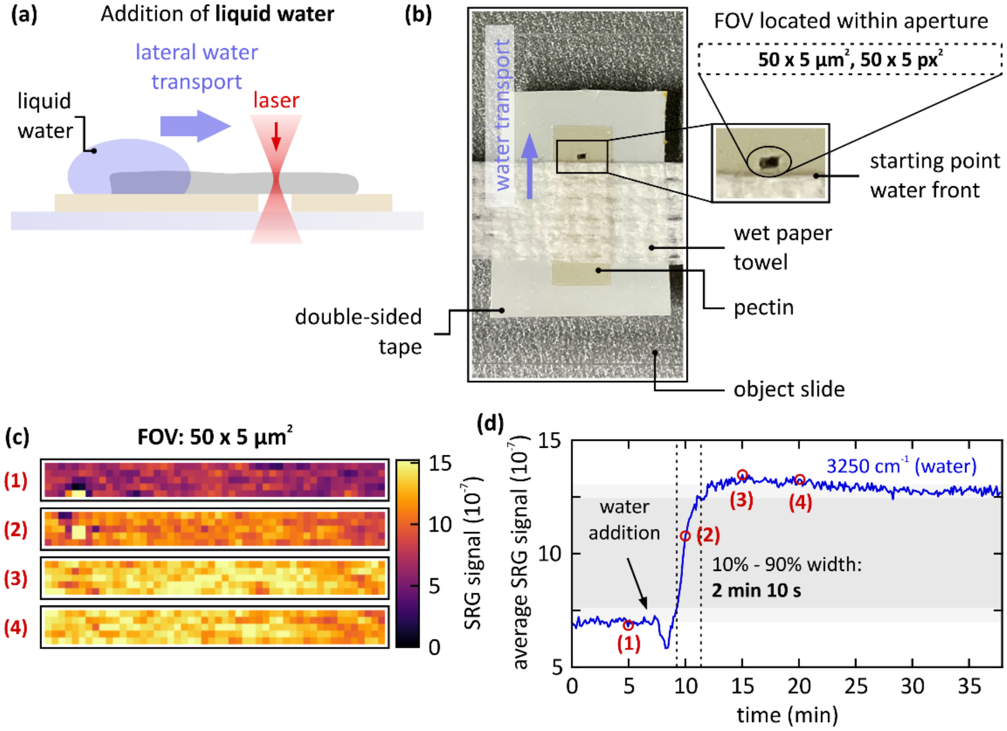

Figure S2. (a) Schematic for measuring the water transport in the pectin, as shown in Fig. 4 of the main manuscript. (b) Close-up photograph of the pectin patch which is glued on double-sided tape. An aperture in the tape enables the SRS signal detection. A wet water towel on top of the pectin polymer delivers a defined water front, which traverses the field of view (transport direction indicated by the blue arrow). (c) Exemplary individual raw data scans, which are obtained at t = 5, 10, 15, and 20 min, as indicated in (d). No structured water channels are visible. Therefore, the water transport takes place either homogeneously on the molecular scale or within small water channels which are well below the diffraction limit. Each data point in (d) corresponds to the average of one entire raw data scan.

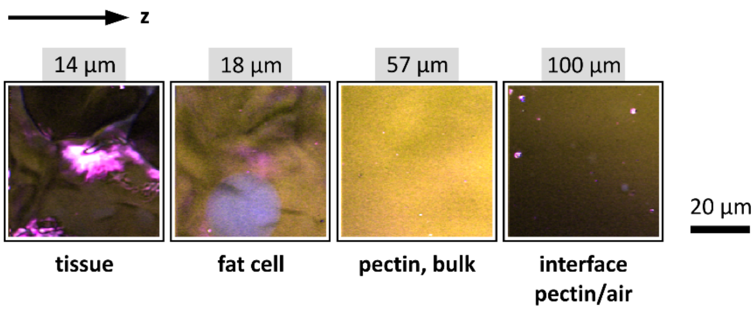

Figure S3. Colorblind-friendly version of the RGB overlays shown in Fig. 5(a) in the main manuscript.

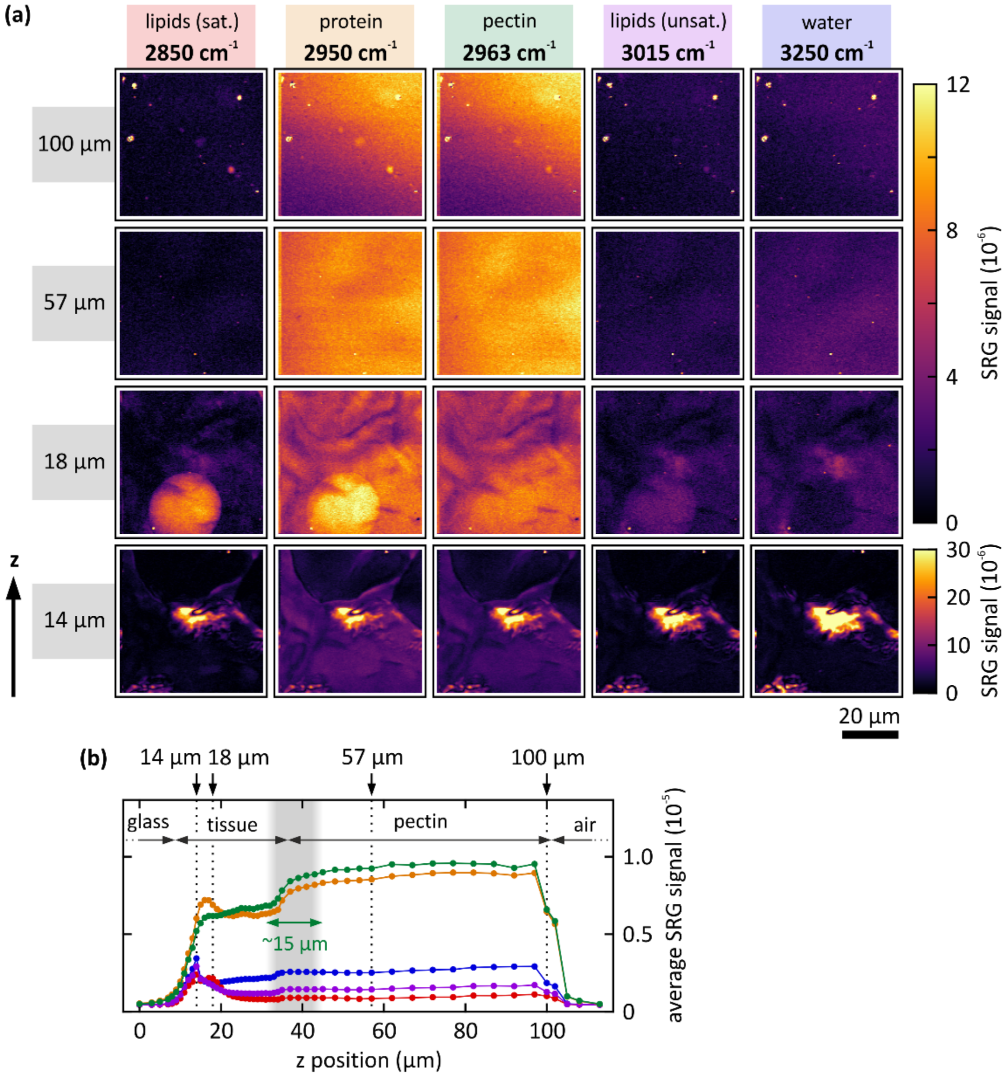

Fig. S4. Two additional Raman bands are shown, i.e., protein at 2950 cm^{-1} and unsaturated lipids at 3015 cm^{-1}. The protein resonance overlaps with the pectin resonance, as their peak positions are separated by only 13 cm^{-1}. (a) A large amount of protein is present in the intestinal tissue at z = 14 µm. The fat cell at z = 18 µm mainly consists of saturated lipids, as evident from the significantly lower signal level in the channel of unsaturated lipids. (b) As expected from their spectral vicinity, the average signal levels along the z-axis of pectin (green) and protein (yellow) are closely correlated. However, in the tissue at z = 15 µm the protein signal exceeds the pectin signal level. Correspondingly, this behaviour is switched in the pectin bulk material. Total acquisition time for the entire data set: 5 h 45 min.

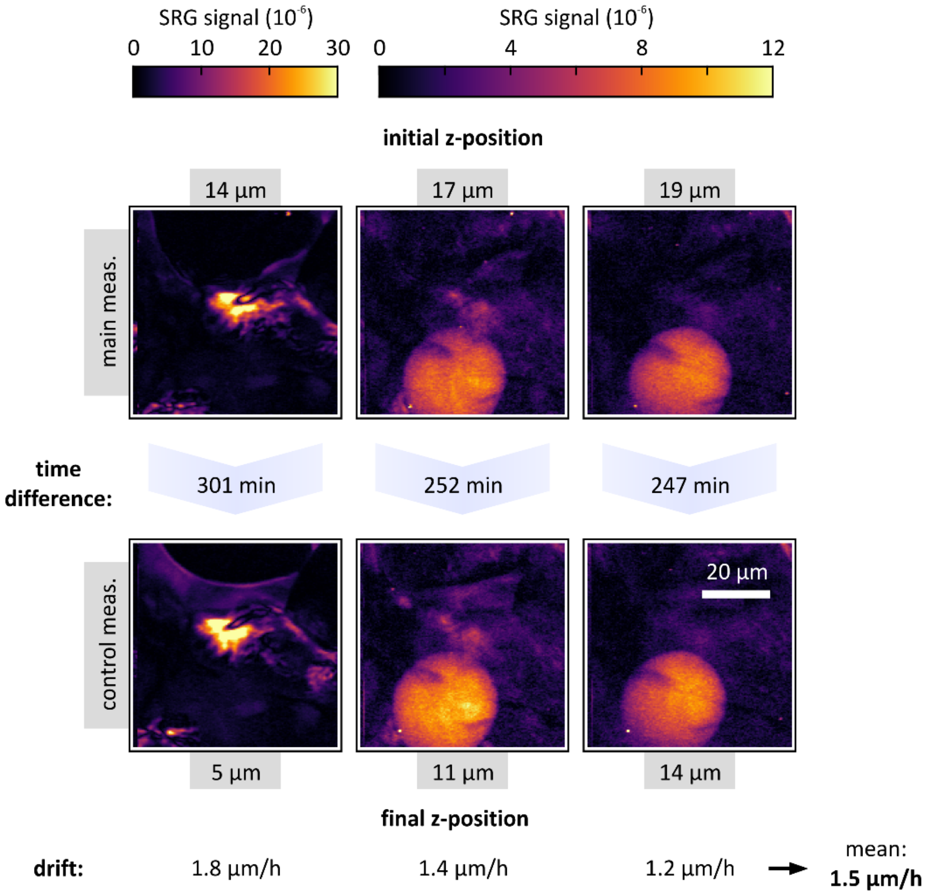

Figure S5. Quantitative analysis of the vertical positioning drift due to sample dehydration. After the main measurement run, three structures within the tissue are located again in order to quantify vertical sample shrinkage. The mean drift amounts to ~1.5 µm/h. All images are acquired at 2850 cm^{-1}.
